# Justice: a key consideration in health policy and systems research ethics

**DOI:** 10.1136/bmjgh-2019-001942

**Published:** 2020-04-08

**Authors:** Bridget Pratt, Verina Wild, Edwine Barasa, Dorcas Kamuya, Lucy Gilson, Tereza Hendl, Sassy Molyneux

**Affiliations:** 1 Centre for Health Equity, School of Population and Global Health, University of Melbourne, Melbourne, Victoria, Australia; 2 Institute of Ethics, History and Theory of Medicine, Ludwig-Maximilians-University, Munich, Germany; 3 Kenya Medical Research Institute (KEMRI) - Wellcome Trust Research Programme, Kilifi, Kenya; 4 Health Policy and Systems Division, School of Public Health and Family Medicine, University of Cape Town, Cape Town, South Africa; 5 Department of Global Health and Development, London School of Hygiene and Tropical Medicine, London, UK; 6 Nuffield Department of Medicine, Oxford University, Oxford, UK

**Keywords:** health services research

## Abstract

Health policy and systems research (HPSR) is increasingly being funded and conducted worldwide. There are currently no specific guidelines or criteria for the ethical review and conduct of HPSR. Academic debates on HPSR ethics in the scholarly literature can inform the development of guidelines. Yet there is a deficiency of academic bioethics work relating to *justice* in HPSR. This gap is especially problematic for a field like HPSR, which can entail studies that intervene in ways affecting the social and health system delivery structures of society. In this paper, we call for interpreting the principle of justice in a more expansive way in developing and reviewing HPSR studies (relative to biomedical research). The principle requires advancing health equity and social justice at population or systems levels. Drawing on the rich justice literature from political philosophy and public health ethics, we propose a set of essential justice considerations to uphold this principle. These considerations are relevant for research funders, researchers, research ethics committees, policymakers, community organisations and others who are active in the HPSR field.

**Summary box**

- There is a deficit of health policy and systems research (HPSR)-specific ethical guidance, particularly in relation to matters of justice.
- We call for interpreting the ethical principle of justice in a more expansive way for HPSR relative to biomedical research.
- Drawing on the rich justice literature from political philosophy and public health ethics, we propose a set of essential justice considerations to uphold this principle.
- These considerations are relevant for research funders, researchers, research ethics committees, policymakers, community organisations and others who are active in the HPSR field.

## Introduction

**Figure 1:**
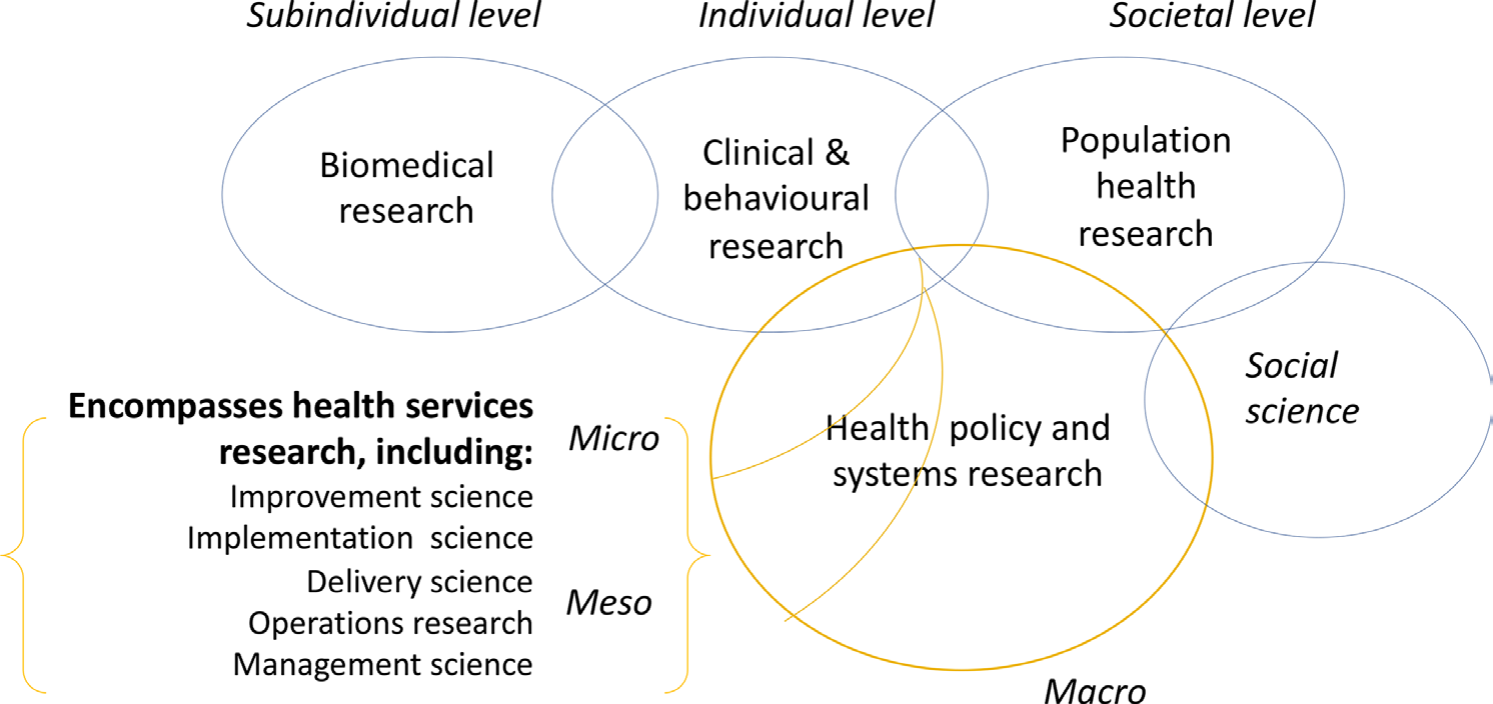
HPSR (adapted from Hoffman *et al*^6^). HPSR, health policy and systems research.

Health policy and systems research (HPSR) is increasingly being funded and conducted worldwide.^1^ In a global context of persistent disparities in access to high-quality health services, rising healthcare costs and with many households facing catastrophic levels of healthcare expenditure, demand for health system strengthening through robust HPSR is rapidly growing.^2 3^ The boundaries, definitions and characteristics of HPSR are still being debated, but emerging consensus is that HPSR is primarily defined by the question it asks rather than its methodological approach. Central foci are the performance of health systems and their subcomponents (hardware: financing, governance, human resources, medical commodities and information systems; and software: power, values and relationships), consideration of how links among the subcomponents shape performance and how to strengthen health system performance over time.^4^ HPSR relies on a wide range of methods that span positivist traditions using fixed research designs, such as economic evaluations, randomised control trials and other epidemiological designs, and relativist traditions using flexible research designs such as qualitative case studies, ethnographic design and participatory action research.^5^ HPSR has strong synergies with research approaches, including implementation science, improvement science, delivery science, operational research and management research (figure 1).^6^

There are currently no specific guidelines or criteria for the ethical review and conduct of HPSR.^7^ For this reason, research ethics committees at most institutions apply well-established biomedical research ethics review criteria and guidelines to HPSR.^8^ These guidelines include criteria related to three prominent principles of biomedical research ethics: respect for persons, beneficence and justice.^9^ This is problematic: while HPSR and traditional biomedical research share many ethical principles and concepts, the two fields differ in numerous ways, including in the ethical issues and considerations that arise.^8 10^ This is arguably especially the case for issues and considerations related to the principle of justice, which has traditionally been understood in distributive terms—as the fair distribution of burdens and benefits—in biomedical research ethics. This is insufficient for HPSR, for which the considerations are much more complex than purely distributive.

HPSR can entail studies that intervene in ways affecting the social and health service delivery structures of society and, thus, have implications for social justice, namely, whether social structures ensure people’s health, well-being and participation. It has often been identified as an essential means to produce the knowledge necessary to reduce health disparities between and within countries,^11 12^ and as having potential to improve our understanding of how to change the odds for marginalised populations to achieve healthy lives.^13^ However, there is also the potential for HPSR to have negative implications for social justice: the focus of HPSR or the way in which it is conducted can inadvertently undermine people’s health or well-being, or increase disparities in access to social and health service delivery structures.

For HPSR, a tailoring of the ethical design and review process is needed, with the WHO arguing there is a ‘compelling need’ for HPSR-specific guidelines and criteria (Luyckx *et al*, p1)[10]. Yet there is a deficiency of academic bioethics work relating to *justice* in HPSR, with the majority of existing scholarship focusing on autonomy and informed consent.^7^ Some justice considerations have been identified for the field,^7 10 14^ but they are not comprehensive. This gap is especially concerning for a field like HPSR, where the knowledge generated can have significant implications for health and social justice at population and systems levels. It is therefore imperative that justice considerations be articulated and discussed specifically, and guidance on addressing them be formulated, to inform the development of HPSR ethics guidelines.

In this paper, we begin by taking the position that the principle of justice itself should be interpreted in a more expansive way for HPSR (relative to biomedical research), in a way that is consistent with the foundational moral commitments of public health. Drawing on the rich justice literature from political philosophy and public health ethics, which has largely not yet informed HPSR ethics, we elaborate on what advancing justice means for the field of HPSR. We then describe what considerations of justice are essential to take into account for HPSR to uphold that principle, not only in HPSR priority setting and funding allocation, but also in reviewing and designing HPSR projects and programmes. These considerations are addressed to funders, researchers, policymakers, practitioners, community organisations, research ethics committees and others who are active in the HPSR field. We conclude by discussing and responding to several possible objections to our proposed justice considerations for HPSR. We further note that our focus on justice should be considered in addition to, and not instead of, adherence to other ethical principles and values in HPSR.

## HPSR’s underlying moral commitment to health and social justice

Much HPSR, especially in low-income and middle-income countries (LMICs), is conducted with the ultimate aim of reducing health disparities between and within countries and enhancing health system performance for those considered disadvantaged and marginalised.^8^ Recent work in bioethics suggests such an aim is necessary to advance health and social justice globally,^15 16^ and advancing justice is consistent with foundational moral commitments for public health research, practice and policy.^17–19^ Upholding justice in HPSR calls for not only achieving a fair share of benefits and burdens for stakeholders in programmes of research, but also advancing health equity and, ultimately, social justice at a population or societal level.

To clarify what advancing justice means for HPSR, a definition of health and social justice is required. While acknowledging definitional controversies within philosophy, a number of points of convergence and commonality do exist. First, theories of justice in health emphasise the fundamental value of health for all, independent from gender, ethnicity, place of birth or residence, social status, political beliefs and religion.^18 20^ Second, multiple theories purport that it is a priority and duty of justice to avert and alleviate disadvantage.^18 20 21^ Powers and Faden, for example, argue that the moral aims of public health are to improve health and other dimensions of well-being, with priority given to the needs of the systematically disadvantaged.^18^ Systematic disadvantage has been defined as being vulnerable to or having large shortfalls on a cluster of dimensions of well-being, including health, security from physical and psychological harm, attachments, self-determination, respect, and sense and imagination.^18 21^ A focus on structural injustices—social norms and institutions that create an unequal playing field—is especially important to identify, avert and alleviate disadvantage.^22 23^

Multiple theories of justice call for bringing disadvantaged individuals and groups up to a ‘sufficient’ level of health and well-being, that is, that which is required for a decent life over a ‘normal’ life span (such as 75 years).^18 24 25^ To attain and maintain a sufficient level of health, individuals are entitled to (among other things) public health and healthcare systems that provide (1) universal/equitable access to quality healthcare services that they need and (2) protection against financial hardship due to out-of-pocket healthcare expenditures through equitable prepayment health financing mechanisms.^25 26^ Access to broader social or structural determinants of health and well-being is necessary as well.^20 25^ In countries worldwide, this encompasses ensuring sufficient health for refugee and migrant populations (among others).^22 27^ In countries with a colonial history, this means ensuring Indigenous health (particularly in regard to access to quality nondiscriminatory healthcare services) and decolonising healthcare systems and broader social structures that shape health.^28 29^ In LMICs, supporting sufficient health and well-being for individuals and groups requires meaningful as opposed to tokenistic capacity strengthening of local and national public health, healthcare and health research systems, as well as in some cases direct support from high-income countries.^16^ The aim is for countries to become capable of ensuring equitable population health and well-being.

A growing number of theories of justice emphasise that, in addition to sufficient health and well-being, a focus should be on agency, participation and epistemic justice aimed at building relational or democratic equality.^30–34^ Agency is the ability to act on behalf of what you have reason to value and entails participating in determining one’s own and society’s actions.^31^ Epistemic justice means giving proper respect to individuals as knowers and sources of information. Democratic participation and epistemic justice constitute a means for citizens to ensure that their needs and interests are raised and reflected in public policies. These theories have recently begun to be applied to health and support robust citizen or community participation in health system decision-making.^35 36^ Theories of justice also identify procedural requirements for decision-making about health matters, which describe how democratic participation should occur. They generally call for relying on deliberative democratic processes and norms, including reasonableness, inclusion, equal voice, accountability and transparency, to achieve just decision-making.^25 37 38^

Finally, theories of justice employ principles to assign specific parties specific responsibilities and obligations of justice. For example, ‘functional requirements’ or ‘capability to act’ principles assign obligations of justice to those who, by their roles and resources, are best positioned to fulfil them.^23 26^ Applying these allocative principles and others to the research context demonstrates that ethical responsibilities of justice fall not only on individual researchers but also on other parties, such as funders, research institutions, ethics review committees and governments.^16 39^

## Essential justice considerations for HPSR

To support researchers and other actors in the HPSR community to give more central emphasis to the principle of justice in HPSR priority setting, funding allocation, design and ethical review, we translate the general facets of health and social justice described previously into specific justice considerations for HPSR. We further identify which HPSR actors bear responsibility to consider them using the capability to act principle (table 1). While the considerations we identify may not be an exhaustive list, they comprise a robust starting point that can be refined and expanded on in the future. Many would also apply to related research approaches, such as implementation science and operational research. The order in which the considerations are presented assumes the following typical sequence of events in research (we recognise variations to this order may occur in practice): priorities are set and funding calls made, and then research teams are assembled and projects designed in response. Only some projects are funded, which undergo ethics review and then, if approved, are implemented.

**Table 1:** Essential justice considerations for HPSR projects and programmes

Essential justice considerations	Who is responsible for considering them
HPSR priority setting	
Do funding platforms and calls prioritise research on equity in health systems and their structural determinants?	Funders during priority setting
Are local actors from LMICs included in making decisions about the topics of HPSR funding platforms and calls?	Funders during priority setting
Do funding platforms and calls require or permit lead applicants from LMICs?	Funders during priority setting
Do funding requirements ensure sufficient funding of local actors and institutions to perform their roles?	Funders during priority setting
Research teams	
Does the research team include local researchers and other local actors from populations involved in the study or at least with deep knowledge about those populations? Will they be included as partners and be part of decision-making throughout the project: from selecting research questions and designing the study to dissemination?	Funders when allocating resources
	Researchers and partners when assembling teams
	Ethics committees when reviewing projects
Are research team members familiar with the sociopolitical historical background of populations involved in the study and the social inequalities they experience?	Researchers and partners when assembling teams
	Ethics committees when reviewing projects
Are any inadequacies in research team composition and representation of local actors, especially marginalised groups and health system actors, recognised and discussed?	Researchers and partners when assembling teams
Are systematic initiatives undertaken for local actors who are part of the research team to strengthen their capacity to conduct independent HPSR?	Researchers and partners when assembling teams
	Ethics committees when reviewing projects
Research questions	
Are local researchers and other local actors on the research team leading or, at a minimum, part of decision-making on the research questions?	Funders when allocating resources
	Researchers and partners when designing projects
	Ethics committees when reviewing projects
Will disadvantaged and marginalised groups or health system actors (or organisations representing them) share decision-making as part of the research team or, at a minimum, be consulted in setting the research questions?	Funders when allocating resources
	Researchers and partners when designing projects
	Ethics committees when reviewing projects
Do the research questions align with the priorities of disadvantaged and marginalised groups or health system actors?	Funders when allocating resources
	Researchers and partners when designing projects
	Ethics committees when reviewing projects
Will answering the research questions create new knowledge of value for equitable health systems?	Funders when allocating resources
	Researchers and partners when designing projects
	Ethics committees when reviewing projects
Research populations	
Does the research population and participants adequately include disadvantaged and marginalised groups and health system actors?	Funders when allocating resources
	Researchers and partners when designing projects
	Ethics committees when reviewing projects
Will research project recruitment be informed by and be respectful of marginalised groups’ past experiences with research and will meaningful engagement be conducted?	Researchers and partners when designing and implementing projects
	Ethics committees when reviewing projects
Identifying and responding to harms	
Will engagement and communication systems be set up that anticipate and keep track of harms generated by HPSR, especially for local actors within the research team, communities and health systems?	Researchers and partners when designing and implementing projects
	Ethics committees when reviewing projects
How will the study team act to minimise anticipated harms and issues that eventuate to disadvantaged and marginalised groups and health system actors while also ensuring that the integrity of the science and the learning—especially about the most vulnerable within systems and communities—is maintained?	Researchers and partners when designing and implementing projects
	Ethics committees when reviewing projects
Research capacity development and health system strengthening	
Do funding platforms require and support strengthening individual and institutional capacity within LMICS to conduct independent HPSR?	Funders during priority setting
How will the project’s design, implementation, publication and data sharing plans strengthen individual and institutional capacity within LMICS to conduct independent HPSR?	Funders when allocating resources
	Researchers and partners when designing projects
	Ethics committees when reviewing projects
How will the study strengthen study participants’ health systems?	Funders when allocating resources
	Researchers and partners when designing projects
	Ethics committees when reviewing projects
How will the project’s design, implementation, publication and data sharing plans minimise the risk of worsening disparities in research capacity?	Funders when allocating resources
	Researchers and partners when designing projects
	Ethics committees when reviewing projects
Creating lasting change	
Does the funding platform require and support knowledge translation of HPSR findings into health and social policy and practice?	Funders during priority-setting
What efforts will be made to maximise positive outcomes or benefits post-study for disadvantaged and marginalised groups and health system actors?	Funders when allocating resources
	Researchers and partners when designing and implementing projects
	Ethics committees when reviewing projects
How are actors with the power to change health and social policies engaged?	Funders when allocating resources
	Researchers and partners when designing and implementing projects
	Ethics committees when reviewing projects
Research funding allocation	
Is funding is allocated to HPSR research teams and projects that have been assembled and designed with justice considerations in mind?	Funders when allocating resources
Do decisions about research funding allocation include local actors from LMICs?	Funders when allocating resources

HPSR, health policy and systems research; LMICs, low-income and middle-income countries.

### HPSR priority setting

For HPSR projects to help alleviate disadvantage and promote relational equality, there must be careful consideration of what research topics are the focus of funding calls, who selects those topics and who is ultimately allocated funding. The priorities set by HPSR funders, who are largely based in high-income countries, strongly determine whether HPSR projects are designed to generate new knowledge to improve healthcare and systems for marginalised groups, communities and health system actors.

Global funding for HPSR is frequently focused on how to expedite the scale-up of priority services. It is less likely to address deeper, more structural factors influencing health system equity.^40^ Theories of justice in health, however, emphasise the importance of generating new knowledge about disparities in access to health services and financial protection, namely, the nature of such inequalities, their causes and how they might be addressed.^16^ When exploring the causes of such disparities, it is especially vital to generate information not only about individuals’ behaviours and health agency (health knowledge, health-seeking skills and beliefs, and effective health decision-making) but also broader structural and social determinants.^20–22 26^ The latter includes the exercise of hidden power (structural and discursive) within and beyond the health system. (Structural power refers to institutional practices (formal rules and procedures) and norms that enhance the capacities or possibilities for action of some and limit those of others. Discursive power encompasses language and concepts that create meanings that lead individuals to think of the world in some ways but not others. By influencing how individuals think about the world, this form of power shapes one’s beliefs, preferences, sense of self and acceptance of the status quo).^41^ The importance of addressing intersecting structural and social determinants of health is due to their potential to cause individuals to fall below or to remain below a sufficient level of health.^18^ A key consideration for HPSR is then: *Do funding platforms and calls prioritise research on equity in health systems and their structural determinants?*

HPSR is frequently entwined with Western funders’ frames and expectations about priorities. Often, at the macro level, Western understanding of global health issues dominate.^42^ Yet many scholars have pointed out that Western conceptualisations of health and well-being are not universally shared or relevant and can, indeed, be in sharp contrast to values and ideas held by some communities and groups.^28 43 44^ Bennett *et al* raise the concern: ‘it is unclear to what extent local actors in LMIC health systems would frame their research concerns in the same way as global stakeholders’.^40^ Inclusion in research decision-making of those who have traditionally been its subjects can bring different, previously under-represented perspectives into research and begin to redress power imbalances.^45 46^ This potentially builds relational equality in HPSR. It is thus essential that the process of setting the topic/focus of funding platforms and calls be inclusive of actors from LMICs who have historically been excluded from such decisions. Funding priorities should not be determined solely by funders and experts from high-income countries. Another important consideration is then: *Are local actors from LMICs included in making decisions about the topics of HPSR funding platforms and calls?* There should be careful consideration of what expertise and which local actors to include and from what sectors. Three important categories of local actors are researchers, marginalised groups (eg, Indigenous and refugee) or health system actors (eg, nurses, community health workers, patients and subnational policymakers), and actors with the power to change the policies and practices that affect them (policymakers, healthcare providers, insurers, civil society organisations and community organisations). The latter category can encompass actors in sectors beyond health, for example, education, sanitation and law.

Kalinga argues that grants should ideally give local actors in LMICs ‘platforms to be authoritative sources of and experts on their cultures and communities’ (Kalinga, p3)[47]. Further considerations for HPSR are then: *Do funding platforms and calls require or permit (co-)lead applicants from LMICs? Do funding requirements ensure sufficient funding of local actors and institutions to perform their roles?*

### Research teams

For HPSR projects to promote health equity, help alleviate disadvantage and promote epistemic justice, it is essential that the composition and functioning of the research team challenge current global injustices in global health research. Research teams must include researchers who understand the sociopolitical and historical background of the populations involved in their studies, including systematically disadvantaged populations. Ensuring inclusion of researchers from (or with deep knowledge of) these populations is an epistemic justice consideration in itself and will likely contribute to research studies being better informed and designed. Ideally, HPSR should be led by researchers from the countries, regions and communities being researched, with strong collaborations with other local actors to bring diverse perspectives and expertise as needed. Involving *under-represented* local actors (ie, marginalised groups and/or actors with health systems, depending on the research focus) also helps to address epistemic injustice and build relational equality.

Crucial considerations about research teams for HPSR are then: *Does the research team include local researchers and other local actors from populations involved in the study or at least with deep knowledge about those populations? Will they be included as partners and decision-makers throughout the project: from selecting research questions and designing the study to dissemination? Are research team members familiar with the sociopolitical and historical background of populations involved in the study and the social inequalities they experience?*

Given the historical and current global structural injustices in health and research systems, it may not always be possible to ensure a genuinely level playing field among research team members throughout the design and conduct of potentially important HPSR. For example, imbalances may arise between non-local researchers and local actors, or between different types of local actors on the research team. Such imbalances require transparency from the outset, and the incorporation of systemic efforts to transform the situation from one of research on or about communities and subpopulations to research by and with their members. Two further considerations for HPSR are then: *Are any inadequacies in research team composition and representation of local actors, especially marginalised groups and health system actors, recognised and discussed? Are systematic initiatives undertaken for local actors who are part of the research team to strengthen their capacity to conduct independent HPSR?* (We discuss capacity strengthening in more detail later in the paper).

### Research questions

For HPSR projects to promote agency and generate knowledge that will help improve health systems for those considered disadvantaged, there must be careful consideration about what research questions are selected and by whom. Many voices, needs and agendas exist within the HPSR field: those of funders, global health actors, health systems researchers, policymakers, healthcare providers and communities. Given the imbalances outlined in previous sections, some voices are often excluded from question setting. LMIC researchers, for example, are often relegated to the role of ‘a glorified field worker’, responsible for providing samples or conducting interviews but excluded from the ‘creative, interesting and scientific’ features of the collaboration.^48^ A key consideration for HPSR is then: *Are local researchers and other local actors on the research team leading or, at a minimum, part of decision-making on the research question(s)?* These will be local to specific HPSR projects and, therefore, will not necessarily be the same as the local actors included in research priority setting and research funding allocation.

Even where the content of research questions comes from local researchers, health system managers or policymakers, the questions prioritised may not align with those articulated by other local actors, especially those considered marginalised and disadvantaged within communities or health systems (This is a particular problem in societies with colonial past and present, with native populations questioning the legitimacy of settler governments, health policies and interventions, and their failure to incorporate Indigenous values and standpoints^28 29^).^46 49^ The community-based participatory research literature highlights a continuum between the ‘utilisation-focused Northern tradition’ and the ‘emancipatory Southern tradition’.^50 51^ The former seeks to produce knowledge that addresses the real-world needs of policymakers and practitioners and facilitate its translation into action.^50^ In the Southern tradition, partnerships are formed with subpopulations considered to be disadvantaged and marginalised. In this tradition, research seeks to identify and transform the root causes or material circumstances that produce and reproduce social disparities and hierarchies; an approach that appears quite pertinent to whether HPSR advances health equity and social justice at the population and systems levels.^50^

Some argue that members of marginalised groups are best positioned to identify the most critical health issues and inequalities they face.^45^ Additional procedural considerations are as follows: *Will disadvantaged and marginalised groups or health system actors (or organisations representing them) share decision-making as part of research teams or, at a minimum, be consulted in setting the research question(s)? How can such processes achieve norms of inclusion, accountability and transparency?* This is consistent with requirements of justice relating to agency, deliberation and participation.^21 32 46^

Contemporary theories of justice explicitly include other elements consistent with the so-called ‘Southern tradition’. They call for focusing attention on the importance of generating new knowledge about disparities within health systems—for example, in access to health services, financial protection, and treatment of health system actors—and exploring their social and structural determinants. For HPSR, this could mean exploring how such determinants interact to create poor access to health services and inadequate financial protection for systematically disadvantaged groups and what interventions can address them. Key considerations for HPSR are then: *Do the research questions align with the priorities of disadvantaged and marginalised groups or health system actors? Will answering the research question(s) create new knowledge of value for equitable health systems?* This calls for HPSR to generate new knowledge about how to achieve fair treatment of marginalised health system actors and/or how to improve access and financial protection for marginalised groups. It is consistent with the requirement of justice to prioritise (or focus on) the needs of those considered disadvantaged.^18 21 34^

### Research populations

For HPSR projects and programmes to help alleviate disadvantage in relation to health, there must be careful consideration about who comprises the research population. This encompasses the selection of research populations *and* the recruitment of individual participants into studies.

According to Cassell and Young, ‘[w]here (HPSR) contributes to the planning of services and policymaking, the voice of the socially excluded may be muffled, and that of the better educated and materially secure, artiﬁcially ampliﬁed’. (p316)^52^ Entire geographical areas or subpopulations that are marginalised or disadvantaged (eg, certain districts or wealth groups and types of health system actors) can be excluded from studies. Within selected research areas or populations, disadvantaged and marginalised groups or health system actors can also be excluded from participating in studies. For example, within host districts, marginalised groups may not be reached by recruitment materials for HPSR studies. Participants in HPSR and emergent learning from studies will then only represent and reflect the experiences of a limited, better-off or more visible subset of the population. This means studies may misrepresent and misunderstand disadvantaged and marginalised groups’ and health system actors’ experiences, or generate knowledge and learnings that do not strongly apply to such subpopulations. In so doing, research processes and outputs may inadvertently exacerbate, rather than reduce, unacceptable differences in experience, health and well-being between and within populations.

A key consideration for HPSR studies is then: *Does the research population and participants adequately include disadvantaged and marginalised groups and health system actors?* ‘Adequate inclusion’ could mean that HPSR studies either focus on particular disadvantaged or marginalised groups or health system actors to produce new knowledge with the potential to transform their situation, or sufficiently include such groups and actors in the research population to ensure that disadvantage and marginalisation are better understood and highlighted. The latter would enable HPSR to generate data that support comparisons between better-off and worst-off groups, and between more and less visible groups. For quantitative studies, adequate inclusion can, for example, produce knowledge of difference or equivalence of health or health system issues across different social or geographical stratifiers, or intersections between several stratifiers. For qualitative studies, it can support, for example, exploration of the lived realities of those groups and health system actors considered disadvantaged or marginalised and help them identify their own abilities and vulnerabilities (including structural drivers behind revealed vulnerabilities inequities).

Working towards adequate inclusion requires recognition that members of some structurally marginalised groups might have good reasons to avoid or be suspicious about participation in research.^46^ Many scholars have emphasised the crucial role of community engagement in building trust between researchers and communities.^45 47 53^ Community engagement can also support inclusive participant recruitment strategies. An important concern for some HPSR studies is then: *Will research project recruitment be informed by and respectful of marginalised groups’ past experiences with research and will meaningful engagement be conducted?* This is consistent with the requirements of justice relating to building relational equality.

### Identifying and responding to harms

To ensure HPSR projects do not exacerbate existing disadvantage and widen disparities in health and well-being requires anticipation and monitoring of negative effects that fall disproportionately on local actors.^15 45 46^ These include medical (physical and psychological), social, economic and political harms.^8^ Here, there is a special interest in hearing about any issues and harms that are anticipated or experienced by those who have the least voice and power within research teams, health systems and communities. Among research teams, unequal power dynamics can prevent local actors, for example, from respecting local social customs and codes.^47^ An ethical consideration for HPSR is then: *Will engagement and communication systems be set up that anticipate and keep track of harms generated by HPSR for local actors who are potentially disadvantaged and marginalised within research teams, health systems and communities?* This is consistent with the requirements of justice relating to epistemic justice and participation. The harms identified for local actors within and external to the research team will likely differ and require different approaches to mitigate or resolve.

To ensure that HPSR projects do not widen existing disparities further entails *acting* on anticipated harms where monitoring processes suggest that HPSR is having negative impacts. Efforts should be taken to develop and implement strategies to minimise anticipated and identified harms. Another ethical consideration for HPSR is then: *How will the study team act to minimise and address anticipated harms and issues that eventuate to disadvantaged and marginalised groups and health system actors while also ensuring that the integrity of the science and the learning—especially about the most vulnerable within systems and communities—is maintained?*

### Research capacity development and health system strengthening

Promoting health of those considered disadvantaged and marginalised requires strengthening in-country capacity to deliver health services and lead essential health research, especially in LMICs. All HPSR projects and programmes in LMICs, whether initiated within countries or by external researchers, should contribute to the sustainable development of health systems and health research systems. Building researchers’ and institutions’ capacities to drive nationally relevant HPSR is a key aspect of achieving that goal. Ethical considerations for HPSR studies include *Do funding platforms require and support strengthening individual and institutional capacity within LMICs to conduct independent HPSR? How will the project’s design, implementation, publications and data sharing plans further those aims? How will the study strengthen study participants’ health systems?*

It is also critical that HPSR does not exacerbate disparities in research capacity and career development, especially between LMIC researchers and high-income country researchers or between non-Indigenous and Indigenous researchers. This could happen, for example, where LMIC or Indigenous researchers’ roles are limited to data collection, rather than contributing to the entire research process, or where these individuals are inappropriately excluded from authorship. It could also happen where data sharing arrangements undermine LMIC or Indigenous researchers’ opportunity to analyse and publish their datasets. Another ethical consideration for HPSR is: *How will the project’s design, implementation, publications and data sharing plans minimise the risk of worsening disparities in research capacity?*

### Creating lasting change

For HPSR to help improve health systems for those considered disadvantaged and marginalised, the knowledge and learning it generates must be used to inform health system reform, public health and social action. Theories of justice contend that ensuring that individuals and groups have sufficient health requires not only getting health policies right but also getting broader health systems and social policies right. Health system reform and public health efforts should, therefore, ideally inform policies and practices in sectors beyond health like housing, education, urban planning and income.^18 20 25^ This requires promoting the translation of HPSR findings into health *and* social policy and practice in ways that help reduce health disparities.^54^ This, in turn, necessitates executing strategies for promoting the use of HPSR findings by local actors, namely, disadvantaged and marginalised groups and health system actors and/or those actors with the power to change policies and practices that affect them. An ethical consideration for HPSR projects is then: *Does the funding platform require and support knowledge translation of HPSR findings into health and social policy and practice? What efforts will be made to maximise positive outcomes or benefits poststudy for disadvantaged and marginalised groups and health system actors? How are actors with the power to change health and social policies engaged?*

### Research funding allocation and ethics review

Whether HPSR helps alleviate disadvantage and builds relational equality is determined by which projects are ultimately awarded funding. Concern has been raised that some donors primarily fund HPSR in LMICs on a narrow set of questions related to service delivery and scale up rather than HPSR that is responsive to local needs or focused on equity.^47 55^ A key consideration is then: *Is funding allocated to HPSR research teams and projects that have been assembled and designed with justice considerations in mind?* To support building relational equality, research funders should include local actors from LMICs on grant review panels and as advisors when making funding decisions. It is thus important to consider: *Do decisions about research funding allocation include local actors from LMICs?*

Prior to implementation, ethics review committees should assess funded projects for many of the proposed justice considerations (table 1). Projects should ideally also be monitored during implementation, with ethics review committees empowered and resourced to step in where justice considerations are being ignored in ways that could exacerbate existing inequities in health and research capacity. Such continuous review would mark a change in practice for many ethics committees worldwide and would require strong, well-trained and supported ethics committees.

## Addressing potential objections

Several objections can be anticipated to our proposed essential considerations of justice for HPSR. Justice, in itself, is a contested term, with various interpretations existing in the philosophy and ethics literatures. This raises the question: how can demands of justice be fulfilled in HPSR if there is no unanimous agreement on the concept of justice in general? Beyond this, from a practical perspective, how can the demands of justice feasibly be achieved if their realisation is highly complex? To address the first matter, we have sought to rely on points of commonality and convergence among theories of justice to identify the proposed justice considerations for HPSR. We further emphasise that justice considerations require an ongoing process of debate and revision, in constant exchange with theorists in the area of health justice, as well as with researchers, policymakers, community organisations and others in the HPSR field. Such dialogue can consider whether the justice considerations are too demanding to achieve in real-world practice and identify what structural changes may be required to facilitate their being upheld. Fomenting and sustaining this dialogue is the responsibility of all HPSR actors as well as ethicists (who may or may not focus on HPSR and thus may or may not see themselves as HPSR actors).

Another related challenge to our proposed considerations of justice is that their implementation might obstruct or slow down the conduct of HPSR at a time when the need for HPSR is widely recognised. A more inclusive study design that aims to explicitly address disadvantaged groups’ needs might require including, for example, migrants who do not speak the language of the host country; individuals living with disability, illiteracy or dyslexia who might need assistance to participate; or geographically remote individuals whose participation is costly and logistically difficult to achieve. This might create higher costs, require more research staff, and slow down studies and learning. Does the goal of achieving their inclusion as a matter of justice justify the increase in resources and time necessary to attain it? We cannot offer a general solution to this problem. How marginalised groups and health system actors can best be included should be decided on a case-by-case basis. Our proposal is certainly not intended to unnecessarily complicate the research process, and every HPSR project will need to weigh up the advantages and disadvantages of different inclusion mechanisms. In so doing, however, it should be recognised that by designing more inclusive research projects, HPSR will most likely produce results that better reflect the heterogeneous reality and thus help develop more responsive and effective health system practices and policies. Thus, for both justice and data validity reasons, it is likely to be valuable to embrace and understand diverse—including historically marginalised—perspectives in study designs.

It could be argued that a considerable amount of HPSR takes place in high-income countries, where disadvantage and health inequity are not as severe as in LMICs. Is it then necessary and appropriate for the proposed considerations of justice to apply to HPSR worldwide or primarily to HPSR in LMICs? We acknowledge that there are settings in which injustices might be particularly stark and where a focus on HPSR’s procedural and substantive contributions to justice is especially needed. Nevertheless, all societies experience disadvantage, stigmatisation and marginalisation, and the gap between the rich and poor is very wide in some high-income countries. We therefore suggest that the proposed justice considerations be taken into account for HPSR in all countries.

Lastly, we acknowledge that tensions may well exist between the procedural and substantive considerations that we identify. For example, research questions identified with the input of disadvantaged and marginalised groups may not always create strong new knowledge for equitable health systems. More broadly, there may be tensions between the proposed justice considerations and ethical considerations reflecting other values in HPSR (eg, solidarity and beneficence). We emphasise the need for additional conceptual and empirical work to explore the nature of these tensions and how they can be navigated.

## Conclusions

We have proposed that the principle of justice should be interpreted more expansively for HPSR than is routinely the case for biomedical research. To uphold this principle, we have identified a set of essential justice considerations to more centrally incorporate justice into HPSR priority setting, funding allocation, design and ethical review. We recognise the large range of study designs and methodological approaches encapsulated in HPSR, and the diversity in their scope and size, and that there are many HPSR projects that already have justice built in as a core concern and approach. By articulating these considerations, we hope to raise the prominence of dimensions of justice as key *ethical* considerations in HPSR across different types of studies. Taking the proposed justice considerations into account should promote those active in the HPSR field to more systematically link projects and programmes to the promotion of health equity and social justice at the population and systems levels.
